# Supplementary material for: A computational framework for resolving the microbiome diversity conundrum
Source: Nat Commun. 2023 Dec 2;14:7977. doi: 10.1038/s41467-023-42768-4 (PMC10693575; doi:10.1038/s41467-023-42768-4)
Supplement: Supplementary file 1 — Supplementary Information [file 41467_2023_42768_MOESM1_ESM.pdf]

Supplementary Information for:

A computational framework for resolving the microbiome diversity conundrum

I. Daybog & O. Kolodny, 2023

Department of ecology, evolution, and behavior, E. Silberman institute for Life Sciences, The Hebrew University of Jerusalem, Israel.

Previous title (as found in online preprints):

Explaining the microbial diversity conundrum: can microbiome composition be crucial for host fitness, while highly divergent among healthy individuals?

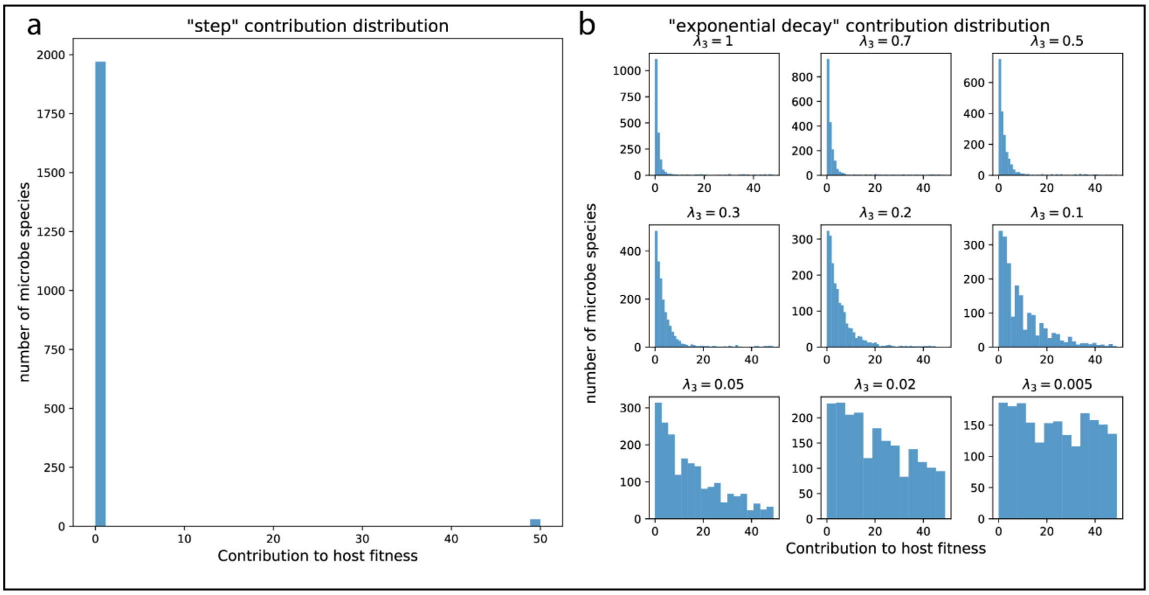

Figure S1. Histograms representing the observed distribution of microbial contributions to host fitness. The histogram data summarizes random repeats of the simulation under various scenarios. **a.** A step background distribution where some microbe species contribute much while others contribute little. **b.** Examples of observed distributions using various exponential decay background distributions with different rate (λ) parameters. $\lambda_3 = 0.005$ is referred to as the "almost uniform distribution" throughout the paper.

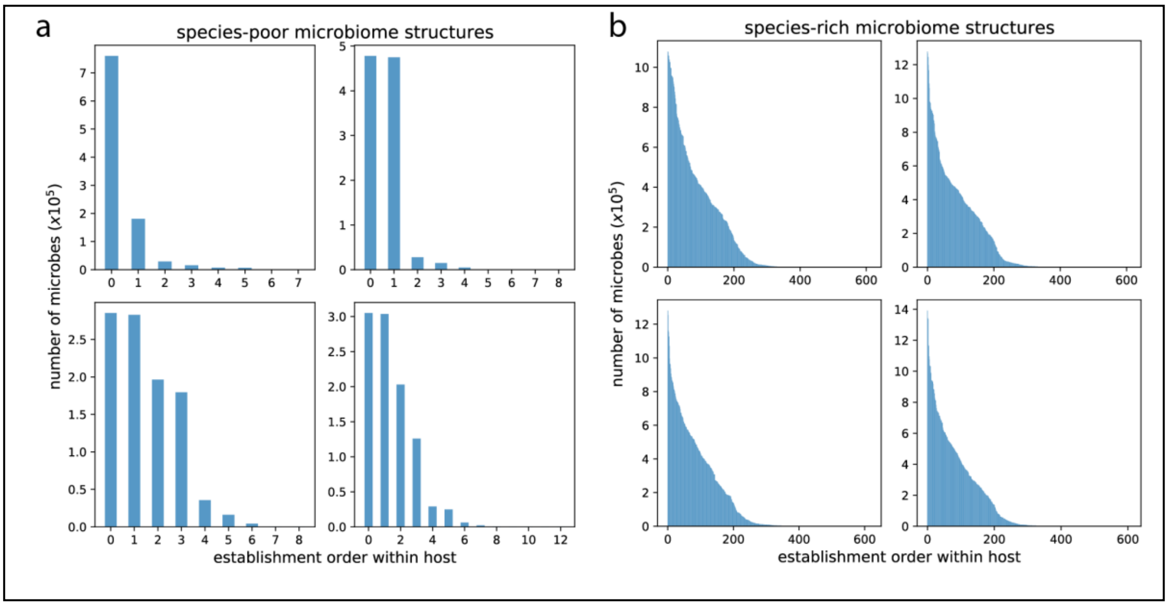

Figure S2. Examples for microbiome structure templates used by the simulation, each one represents a different host. The x-axis sorts the different microbial species according to their order of establishment within the host, and the y-axis shows their final abundance within the host. **a.** Examples of species-poor microbiome configurations. **b.** Examples of species-rich microbiome configurations.

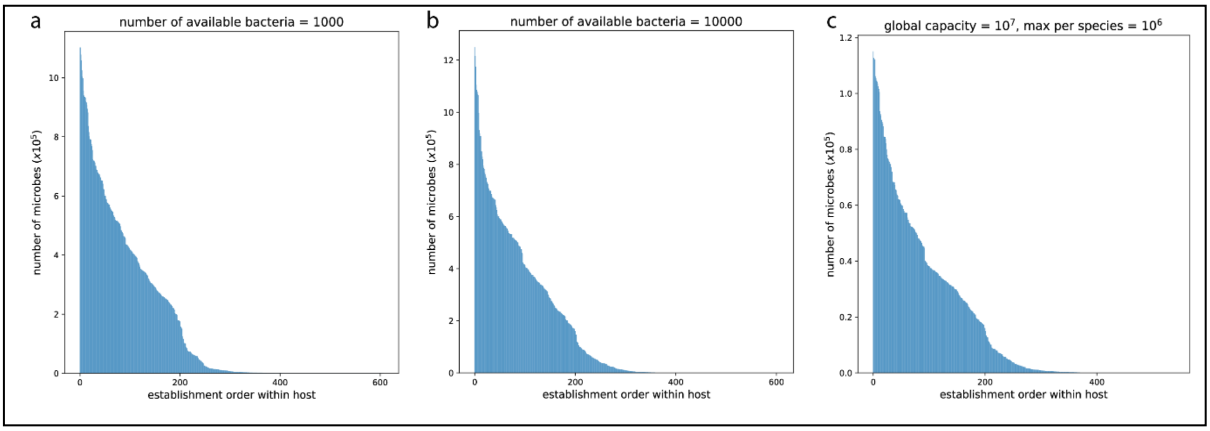

Figure S3. Examples of species-rich microbiome structure templates, generated with altered parameters relative to those used to generate the results in the main text. The main text considers microbiomes structured with 2000 available bacterial species to sample from, a global carrying capacity of 10^8 and a per-species carrying capacity of 10^7 (See fig S2). These are fairly arbitrary choices, and the results are not sensitive to them; the reasoning behind the choice of an environmental pool of 2000 species was that in reality there is high environmental diversity that can be sampled (thousands to tens of thousands of species), but the vast majority of species in the environment are extremely limited in their ability to stably colonize a certain host (e.g. human) gut, and can thus be ignored. Here we explore several additional parameter combinations: **a.** The number of available species of bacteria was reduced to 1000. **b.** The number of available species of bacteria was increased to 10,000. **c.** The global carrying capacity was reduced to 10^7 and the per species capacity was reduced to 10^6.

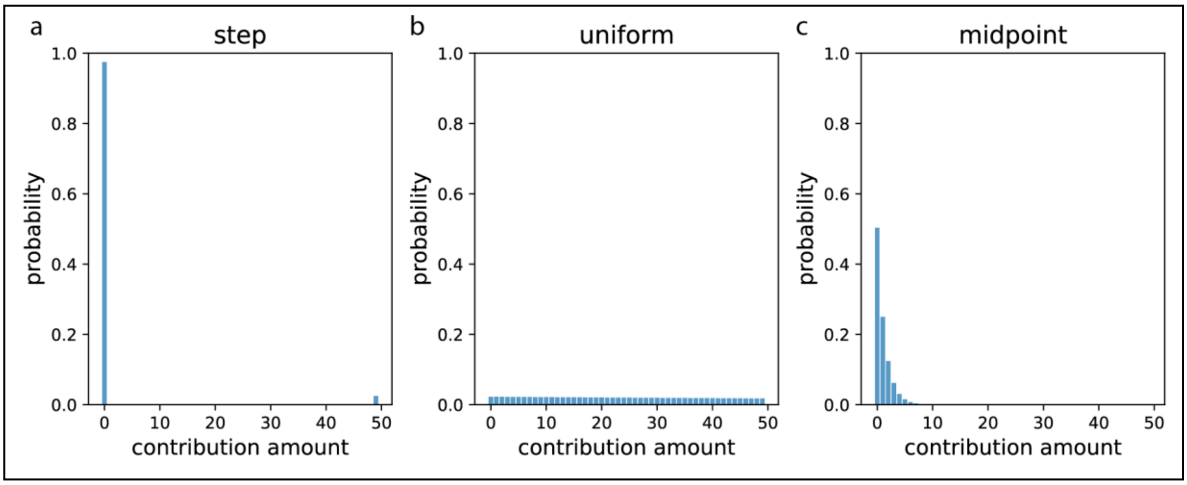

Figure S4. PDF of background distributions for microbial contribution to host fitness. **a.** A step distribution. **b.** An exponential decaying distribution with $\lambda_3 = 0.005$, "almost uniform'. **c.** An exponential decaying distribution with $\lambda_3 = 0.7$, a midpoint between the two other distributions.

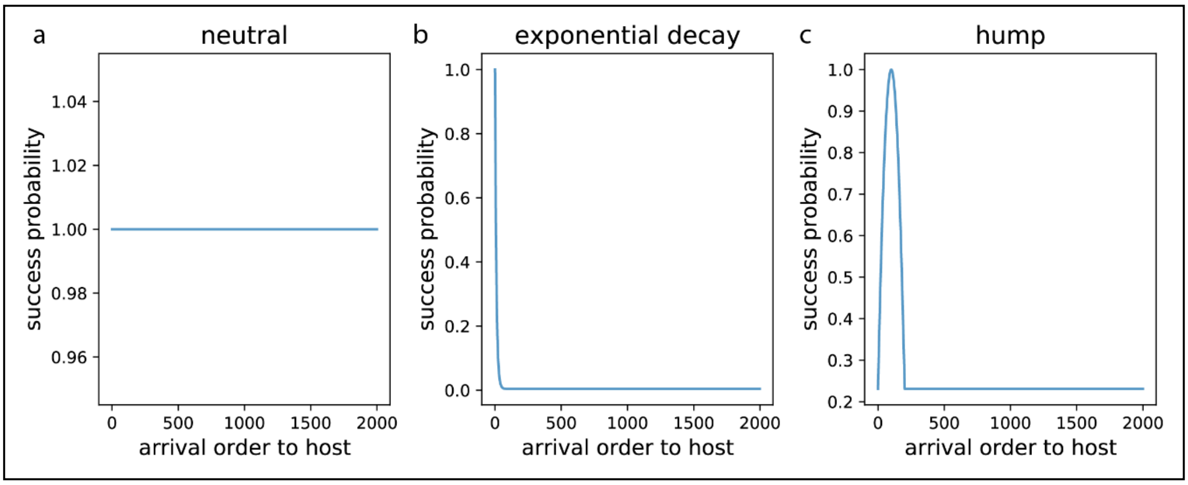

Figure S5. Success probabilities of microbial establishment events according to the arrival index of the microbe species to the host. **a.** a neutral scenario where the establishment within the host is always successful. **b.** a decaying exponential establishment probability, where the first microbe species to arrive have a larger chance for a successful establishment. **c.** A hump shaped establishment probability, where the establishment is most likely to succeed after several pioneer species were already established within the host.

Table S1a. The full list of parameters used by the model to generate the species-rich microbiome configurations.

**Parameter**	**Symbol**	**Value**
Waiting times exponential rate	λ_1	0.7
Hump scaling parabola - a	a	-1.25
Hump scaling parabola - b	b	-2.5
Hump scaling parabola - c	c	-0.5
Hump scaling parabola – peak index	p	100
Hump scaling parabola – scaler	H_s	0.3
Hump scaling parabola – minimal probability	H_m	0.25
Logistic growth – steepness	k	0.005
Logistic growth – midpoint	m	8000
Single taxon carrying capacity	C_s	10^7
Global host carrying capacity	C_g	10^8

Table S1b. The full list of parameters used by the model to generate the species-poor microbiome configurations.

**Parameter**	**Symbol**	**Value**
Waiting times exponential rate	λ_1	0.7
Exponential decay scaling – rate	λ_2	55
Exponential decay scaling – scaler	E_s	0.004
Logistic growth – steepness	k	0.005
Logistic growth – midpoint	m	8000
Single taxon carrying capacity	C_s	$10^{5.9}$
Global host carrying capacity	C_g	10^6

The parameter values (**Tables S1a-S1b**) were chosen such that they would lead to configurations that seem realistic in their structure in comparison to observed host-associated microbiomes. The two categories presented and discussed here, of species-rich and species-poor microbiomes, reflect a difference in a feature of the microbiome configurations that we highlight as influencing the microbiomes' ability to influence selection dynamics among hosts; these and other model results are robust to changes in other parameters that are involved in the configurations' production (see Figures S3, S9-S10, and main text).

We did not attempt to choose parameters that would represent the specific microbiome of any particular host species. The values of carrying capacity for microbiome species and the overall communities we chose are on the order of magnitude that is conceivable for those reported from various body sites of vertebrate and insect hosts, while keeping in mind that within each of these categories there are species that differ from one another by several orders of magnitude in the size of the habitat that they provide to their microbiomes; consider, for example, the gut of a young killifish compared to that of a ruminant. Some estimated values that have been provided in the literature in recent years support our parameter choices as reasonable, e.g. (1–5).

Table S2. The full list of parameters used by the model to generate the background distributions for the microbes' contributions to their host's fitness.

**Parameter**	**Symbol**	**Step value**	**Exponential decay value**
Minimal contribution	C_{min}	0.005	
Maximal contribution	C_{max}	50	
Step size / Rate	λ_3	30	1, 0.7, 0.5, 0.3, 0.2, 0.1 0.05, 0.02, 0.005

In the scenario where microbes' contributions are described by a step function, some microbes contribute C_{min} and others contribute C_{max}. In the case that was used for demonstration of this scenario, 30 microbial species provided the high contribution and the remaining 1970 species provided the low contribution (hence λ_3 = 30). In the scenario in which the contributions are described by an exponential decay function, C_{min} and C_{max} represent the highest and lowest value that the function takes, and the rate parameter λ_3 describes the functions rate of decay.

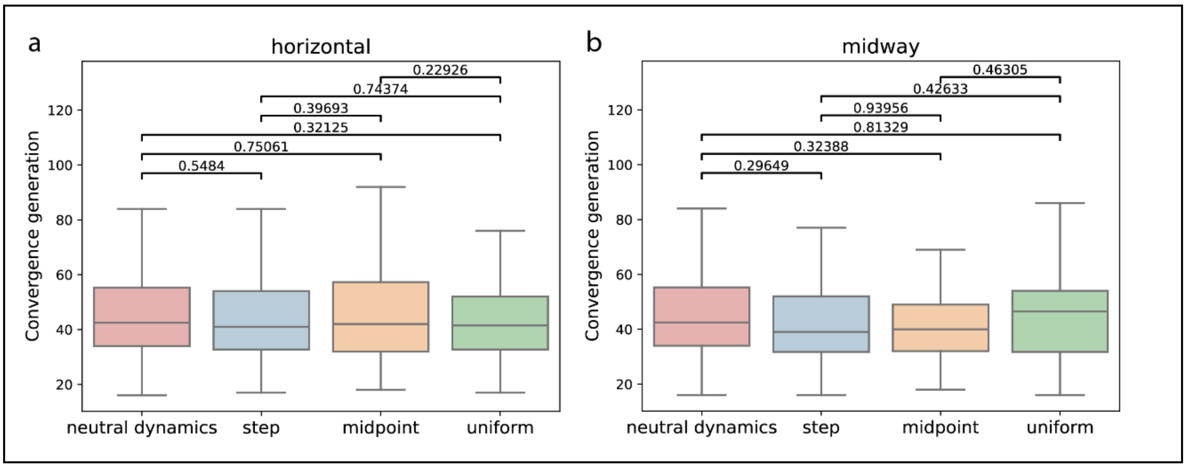

Figure S6. Influence of different distributions of microbes' contributions to host fitness on the number of generations it took for all existing hosts in the population to share a common ancestor, in a species-rich microbiome scenario, across 100 repetitions of the stochastic simulation. **a.** under purely horizontal transmission. **b.** under a midpoint (50% vertical, 50% horizontal) transmission. Neutral scenario without microbiome effect (red), uniform (green), step (blue) and a midpoint between the two (orange).

Figure S6 depicts the influence of the contributions to fitness of the microbes on selection dynamics under non-vertical microbiome transmission schemes. When the microbiome is transferred only horizontally, it has no effect on the hosts' selection dynamics. The same is true in these conditions when transmission is a 50%-50% combination of horizontal and vertical transmission.

None of the different distributions of microbial contributions (Fig. S6) leads to a significant change in the number of generations required until lineage coalescence. This suggests that in our framework, the combination of non-purely-horizontal transmission with a species-rich microbiome leads to a selectively neutral or near-neutral dynamic.

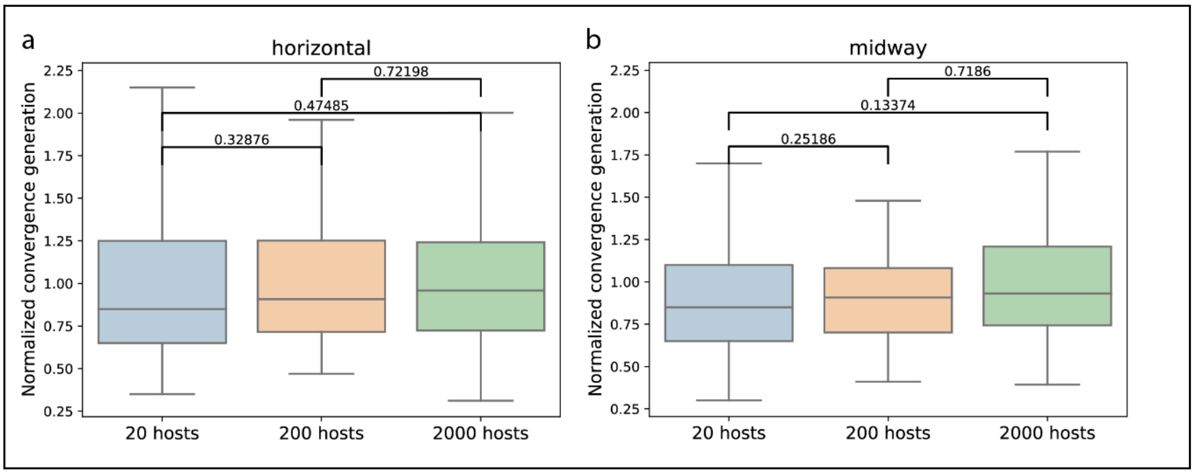

Figure S7. Influence of host population size on the number of generations it took for all existing hosts in the population to share a common ancestor divided by the populations size, across 100 repetitions of the stochastic simulation. **a.** under purely horizontal transmission. **b.** under a midpoint (50% vertical, 50% horizontal) transmission. 20 hosts (blue), 200 hosts (orange) and 2000 hosts (green).

Figure S7 depicts the non-significant differences in time to coalescence when using different population sizes of hosts and non-purely-horizontal transmission of microbes. This suggests that the non-vertical transmission poses a major challenge for selection among hosts to act effectively in response to differences in the microbiome.

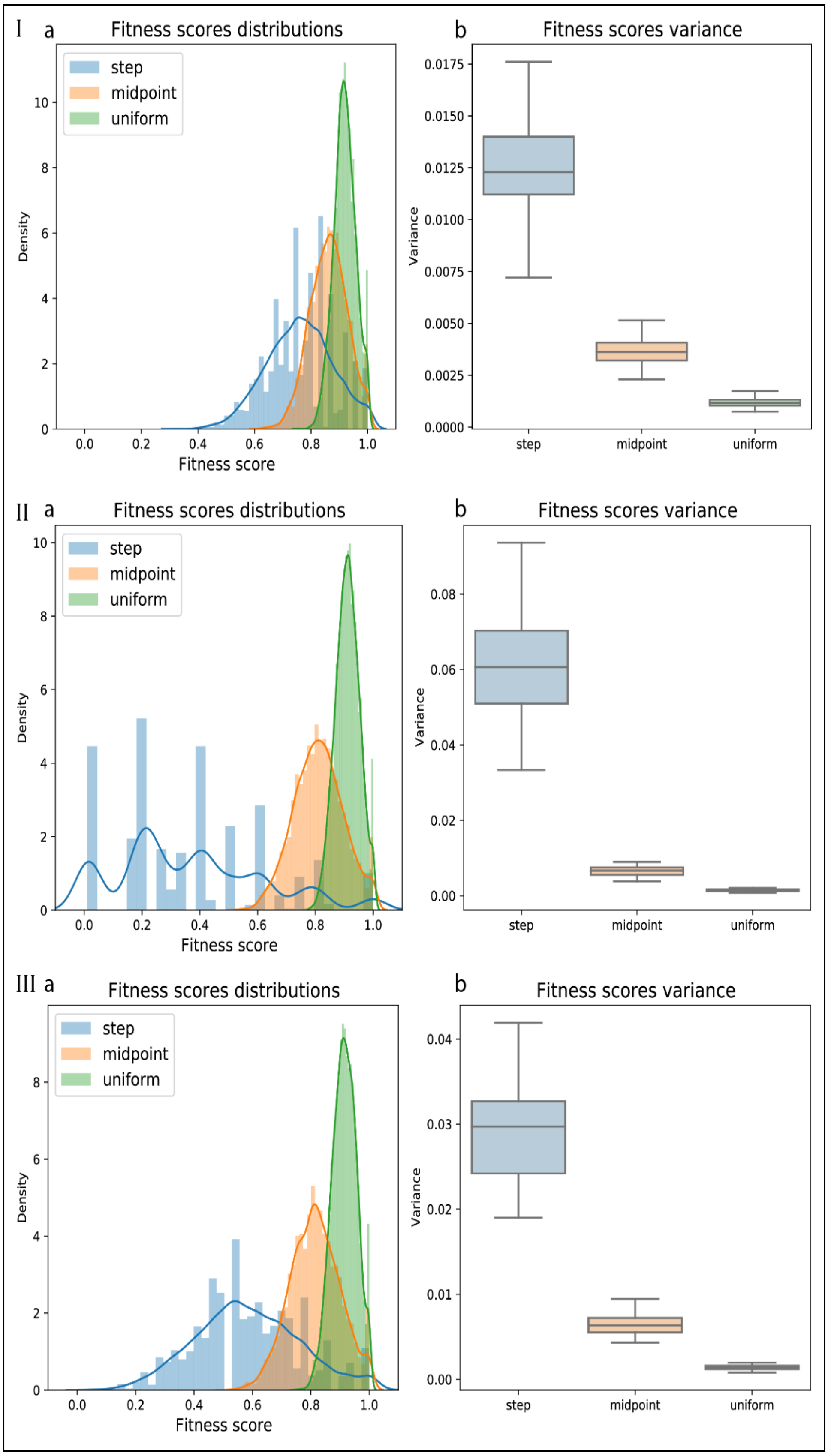

Figure S8. Influence of changing key parameters in the process of generating the microbiome structures within the hosts on the observed results discussed in the main text (See Fig. 6). **I**. Lowering the number of available microbial species to 1000 (vs. 2000 in the main text). **II**. Increasing the number of available microbial species to sample from to 10000 (vs. 2000 in the main text). **III**. Reducing the global microbial capacity of hosts to 10^7 and the maximal capacity per species to 10^6 (vs. 10^8 and 10^7 in the main text respectively). In I-III, panel A depicts the distribution of fitness scores, and panel B depicts their variance.

In order to corroborate that the results presented in the main text are not the outcome of the specific parameters that were chosen, we ran further simulations with different parameter values (**Figure S8**). We ran simulations for three microbiome configurations under the main scenario of interest, in which the ability to drive selection in host populations depends on the distribution of the microbial contribution to the fitness of their hosts. The first two parameter combinations included changes to the number of available microbial taxa to sample from, and the third enforced a lower number of bacteria in each host and a lower maximum capacity per microbial species. We find that in all three parameter combinations the variance in fitness among individuals in the first generation was the same as in the results presented in the main text – where a less uniform distribution leads to higher variance (Fig. 6, Fig S9).

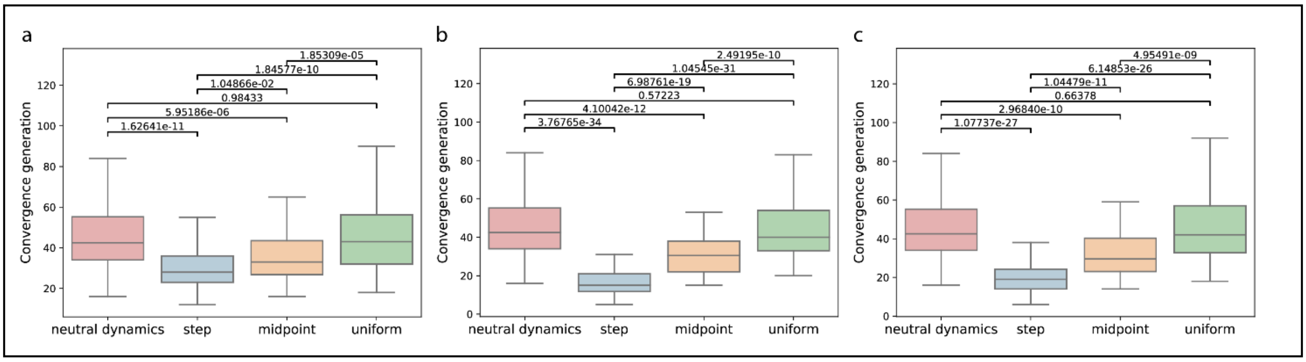

Figure S9. Influence of changing key parameters in the process of generating the microbiome structures within the hosts on the observed results discussed in the main text (See Fig. 7). **a**. Lowering the number of available bacteria species to sample from to 1000 (vs. 2000 in the main text). **b**. Increasing the number of available bacteria species to sample from to 10000 (vs. 2000 in the main text). **c**. Reducing the global microbial capacity of hosts to 10^7 and the maximal capacity per species to 10^6 (vs. 10^8 and 10^7 in the main text respectively).

The times to lineage coalescence in the parameter combinations explored here (**Figure S9**) are also similar to the main text: we find that the ability of the microbiome to influence selection processes in the host population is increased when microbial species differ more significantly in the extent to which they contribute to their hosts (Fig. 7, Fig S10).

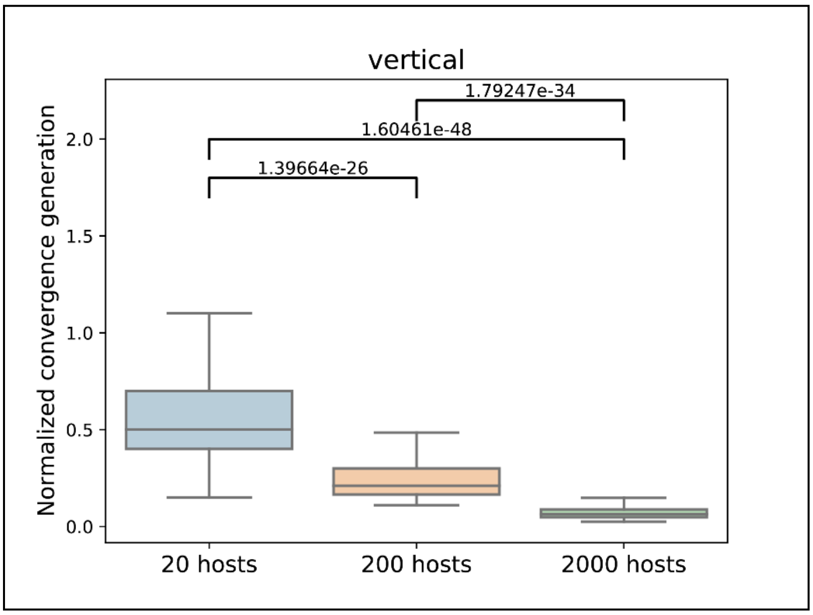

Figure S10. Influence of host population size on the number of generations it took for all existing hosts in the population to share a common ancestor divided by the populations size, under purely vertical transmission, across 100 repetitions using the species-poor microbiome configuration. 20 hosts (blue), 200 hosts (orange) and 2000 hosts (green).

Figure S10 presents the influence of population size on the ability of the microbiome to impact selection processes in populations of hosts with a species-poor microbiome structure, complementing the results shown in the main text for a species-rich microbiome. Not unexpectedly, in this scenario – where the variation in microbiome-mediated fitness between lineages is large – selection occurs in all population sizes, and its efficacy is increased as population size increases.

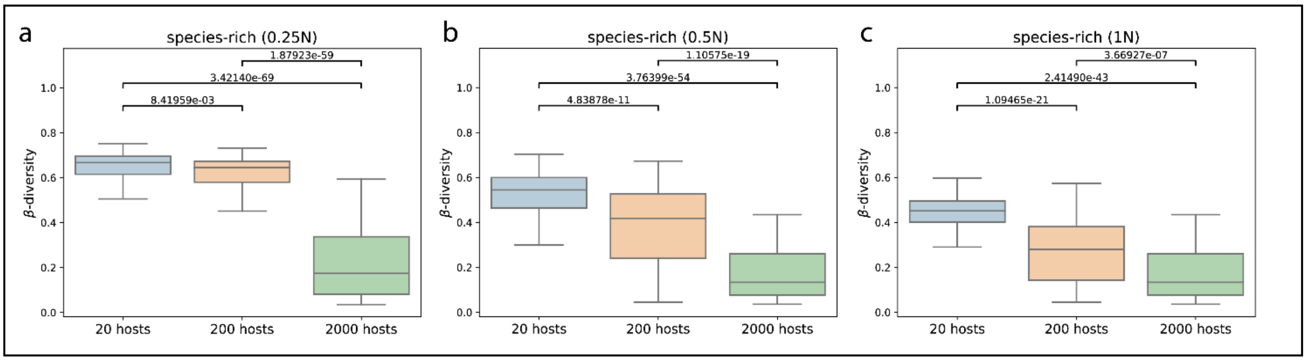

Figure S11. Population β-diversity at different generations in relation to the number of hosts in the population, across 100 repetitions using a species-rich microbiome configuration with vertical transmission. 20 hosts (blue), 200 hosts (orange) and 2000 hosts (green). **a.** At a generation equals to 25% of the population size (i.e generation 5, 50, 500 for the populations of 20, 200, 2000 hosts respectively. **b.** At a generation equals to 50% of the population size. **c.** At a generation equals to the population size. (In runs that ended prior to the required generation, the last generation was taken).

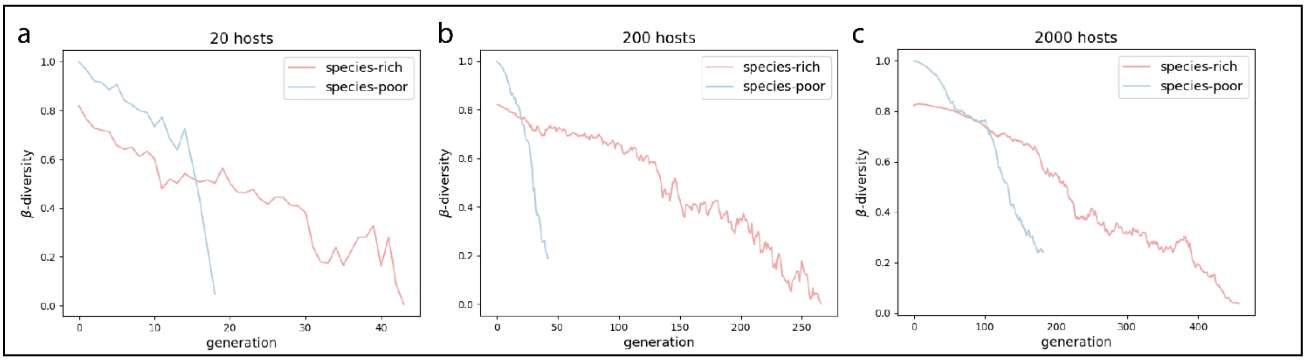

Figure S12. Population β-diversity of representative single runs of the simulation that were run until only one parental lineage was left, comparing populations with varying sizes of hosts with species-rich microbial configurations (red), and species-poor microbial configuration (blue). **a.** population of 20 hosts. **b.** population of 200 hosts. **c.** population of 200 hosts.

β-diversity changes over time along simulation runs (**Figure S11, S12**): it is initially high in all cases, as microbiome compositions are determined in the beginning of each run in a stochastic neutral process (see main text). In the runs with parameter combinations that facilitate effective selection on microbially-mediated fitness (see above and main text), the selection on hosts rapidly reduces the number of lineages in the population, respectively leading to a reduction in β-diversity. This process is most salient in runs with purely vertical transmission as shown here, and where selection on microbially-mediated fitness of hosts is most effective. We focus on this scenario, as in this study we set out to explore the possible underpinnings of cases in which β-diversity remains high even though host fitness is determined purely by its microbiome (the hardest-to-explain scenario). In this respect, the cases where population size is small, rendering selection inefficient and effectively setting the system to be in the near-neutral regime of selection, are the most interesting among those shown in Figures S12-S13, as they offer a possible solution to the β-diversity conundrum. In the simulations shown, for tractability and simplicity, repeated introduction of microbes from the environment is not considered – although this scenario is included in the currently available version of our framework, offering the option of future exploration of this process and its interaction with those that we have focused on. The absence of re-introduction of microbial diversity from the environment leads to a deterministic eventual decrease in the β-diversity, as the number of lineages in the population decreases – even as a result of neutral coalescence. In a more realistic scenario, where microbial transmission is not purely vertical, we suggest that merely neutral coalescence is unlikely to drive β-diversity to low values, and that – as suggested in the discussion in the main text – low β-diversity would be found only when selection on microbially-mediated fitness among hosts is effective.

References

1. R. Sender, S. Fuchs, R. Milo, Revised estimates for the number of human and bacteria cells in the body. *PLoS Biol.* **14**, e1002533 (2016).
2. B. van Schooten, F. Godoy-Vitorino, W. O. McMillan, R. Papa, Conserved microbiota among young Heliconius butterfly species. *PeerJ* **6**, e5502 (2018).
3. S. M. Rudman, *et al.*, Microbiome composition shapes rapid genomic adaptation of Drosophila melanogaster. *Proc. Natl. Acad. Sci.* **116**, 20025–20032 (2019).
4. I. Nasidze, J. Li, D. Quinque, K. Tang, M. Stoneking, Global diversity in the human salivary microbiome. *Genome Res.* **19**, 636–643 (2009).
5. S. K. Ben Shabat, *et al.*, Specific microbiome-dependent mechanisms underlie the energy harvest efficiency of ruminants. *ISME J.* **10**, 2958–2972 (2016).
